# Low-Loss Dual-Band Transparency Metamaterial with Toroidal Dipole

**DOI:** 10.3390/ma15145013

**Published:** 2022-07-19

**Authors:** Tianyu Xiang, Tao Lei, Ting Chen, Zhaoyang Shen, Jing Zhang

**Affiliations:** 1 School of Big Data and Computer Science, Guizhou Normal University, Guiyang 550003, China; leitao2003101@163.com (T.L.); 20010230631@gznu.edu.cn (T.C.); weiwei_yangyang@163.com (J.Z.); 2 State Key Laboratory of Millimeter Waves, Southeast University, Nanjing 210096, China; 3 Hubei Key Laboratory of Intelligent Vision Based Monitoring for Hydroelectric Engineering, College of Computer and Information Technology, China Three Gorges University, Yichang 443005, China; shenzhaoyang@ctgu.edu.cn

**Keywords:** toroidal dipole, metamaterial, low-loss, sensing, slow light

## Abstract

In this paper, a low-loss toroidal dipole metamaterial composed of four metal split ring resonators is proposed and verified at microwave range. Dual-band Fano resonances could be excited by normal incident electromagnetic waves at 6 GHz and 7.23 GHz. Analysis of the current distribution at the resonance frequency and the scattered power of multipoles shows that both Fano resonances derive from the predominant novel toroidal dipole. The simulation results exhibit that the sensitivity to refractive index of the analyte is 1.56 GHz/RIU and 1.8 GHz/RIU. Meanwhile, the group delay at two Fano peaks can reach to 11.38 ns and 12.85 ns, which means the presented toroidal metamaterial has significant slow light effects. The proposed dual-band toroidal dipole metamaterial may offer a new path for designing ultra-sensitive sensors, filters, modulators, slow light devices, and so on.

## 1. Introduction

The electromagnetic multipole expansion of classical electrodynamics contains only the electric and magnetic multipole families, while the toroidal multipole family, as an important part, has long been considered as a correction [1]. The toroidal dipole derived from the current that flows along the meridians of the torus is the fundamental and primitive member of the toroidal multipole family, which was proposed as an explanation of the parity violation by Zel’dovich [2]. However, the electromagnetic response of the toroidal dipole in natural materials is usually masked by much stronger electric and magnetic dipoles. In order to clarify the interesting phenomenon of the toroidal dipole, it is essential to suppress the electric and magnetic multipoles and, at the same time, enhance the toroidal dipole response by a rational method.

Electromagnetic metamaterial is an artificial subwavelength structure, which can manipulate electromagnetic waves by controlling the arrangement and picture of the metamolecule [3,4,5,6,7]. Metamaterial has many special phenomena including negative refraction, superlensing, cloaking, and slow light effects that do not exist in natural materials. In 2010, Zheludev et al., presented a three-dimensional metamaterial to suppress traditional multipoles and observe the toroidal dipole response [8]. This is the first time that the toroidal dipole was experimentally verified. Since then, much research on the dynamic toroidal dipole usually with low-loss and high field concentration has been carried out, such as electromagnetic-induced transparency [9], absorption [10], circular dichroism [11], sensing [12,13], optical force [14], and so on [15,16,17,18,19,20,21,22].

The Fano resonance caused by the interference and coupling between the narrow discrete mode and broad spectrum has been used in many studies [23,24,25,26]. In recent years, the toroidal-dipole-supported Fano resonance has been implemented to concentrate the electromagnetic field in a small region of the substrate [27,28,29,30,31,32,33,34,35]. Gupta et al., proposed a two-dimensional metamaterial to acquire a Fano resonance derived from the toroidal dipole. The interaction between light and matter has been enhanced on account of the strong local field characteristics of the toroidal dipole, and the toroidal sensing was accomplished at terahertz frequency [36]. Fan et al., achieved Fano resonance with high *Q*-factor based on toroidal excitation in a planar metamaterial at microwave range [37]. Liu et al., presented a Fano resonance metamaterial based on the destructive interference between toroidal and electric dipole and the toroidal Fano resonance was flexibly tuned by graphene at the gap [38]. In published papers about Fano resonance, usually only one resonance is induced by the toroidal mode in metal metamaterial. Meanwhile, it is of great significance to realize dual-band Fano resonance both caused by toroidal dipole to improve the performance for analyte detection in sensor applications and increase the frequency band of slow light effects within the microwave range.

In this paper, a two-dimensional subwavelength structure with dual-band Fano resonances is presented and verified. The metamolecule of toroidal metamaterial consists of two pairs of inner- and outer-mirrored metal split ring resonators. Two toroidal-caused Fano resonances are excited under the illumination of the plane electromagnetic waves along the y-polarization. The analysis of the current density distribution and the multipole scattered power at the resonance frequency shows that both Fano resonances are the toroidal dipole response. Due to the strong local field property of the toroidal dipole, the structure has high sensitivity, low-loss characteristics and slow light effects, which are proved by the linear fit of the frequency shift, the imaginary part of the refractive index, and group delay. This is of great significance for the application of metamaterial in ultra-sensitive sensors and low-loss slow light devices.

## 2. Design and Fabrication

**Figure 1:**
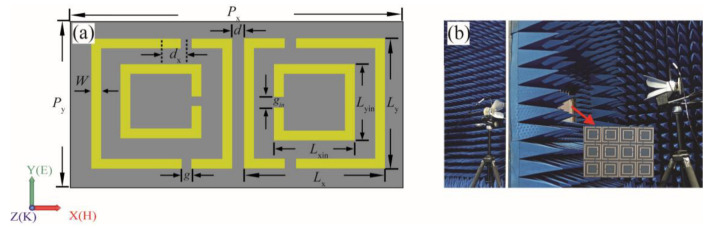
(**a**) The schematic of the metamolecule with structural parameters; (**b**) a photograph of the experimental environment and partial view of the sample.

As shown in the diagram of the metamolecule in Figure 1a, each unit cell is composed of two pairs of mirrored metal split ring resonators (SRRs) on the dielectric substrate. The metal resonators and substrate material are copper and F_4_B (*ε*_γ_ = 2.65) with a thickness of 1.5 mm. The parameters of the outer SRRs (OSRRs) are: *L*_x_ = *L*_y_ = 7 mm, with a gap of *g* = 0.5 mm, the arm width of the resonators *W* = 0.3 mm, and the distance away from the center of the resonator *d*_x_ = 1 mm. A parameter *d* = 0.5 mm is introduced between the mirrored OSRRs to separate the two resonant rings. For the inner SRRs (ISRRs), the parameters are *L*_xin_ = *L*_yin_ = 4.4 mm, and a split-gap size of *g*_in_ = 0.4 mm. The dimension of the unit cell is *P*_x_ × *P*_y_ (15 mm × 8 mm).

Using printed circuit board (PCB) technology, the sample of the whole structure with an overall size of 240 mm× 256 mm is fabricated by 16 × 32 metamolecules arranged periodically along the x- and y-axes, and a partial view of the toroidal subwavelength structure is displayed in Figure 1b. The electromagnetic parameters of the proposed toroidal metamaterial are calculated by the commercial simulation software CST Studio Suite (CST Studio Suite 2018, Computer Simulation Technology AG, Darmstadt, Germany). Open boundary is used in the z-axis, and the periodic boundary conditions are set in the x- and y-axes. The toroidal metamaterial is normally irradiated by the y-polarized planar electromagnetic waves along the z-direction. In addition, the vector network analyzer (Agilent PNA E8362B, Agilent Technologies Inc., Palo Alto, Santa Clara, CA, USA) and dual-ridge horn antennas (Nanjing Guanjun Technology Co., Ltd., Nanjing, China) are used in an anechoic chamber to verify the transmission coefficients of the structure.

## 3. Analysis and Discussion

**Figure 2:**
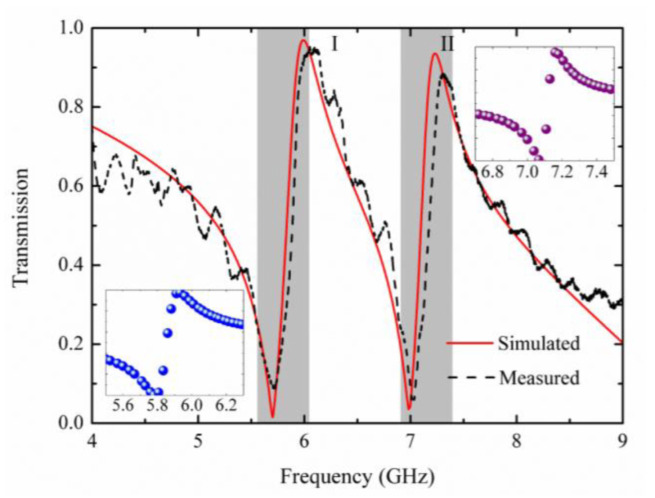
The transmission characteristics of the proposed metamaterial, the simulated (solid red line) and experimental (dashed black line) results. The fitting results of two Fano resonances shown on the left and right inset, respectively.

The simulated and measured spectral lines of the proposed metamaterial are shown in Figure 2. By observing the simulated result, two Fano resonances are seen at 6 GHz and 7.23 GHz corresponding to the transmission peaks at 0.97 and 0.94. The measured result exhibits the clear dual-band asymmetrical Fano resonances as the dashed black spectral line shown in Figure 2, which almost agree with the simulation result. The slight difference between the simulated and measured results might be caused by the fabrication tolerance.

The dual-Fano resonances could be explained by the interference between narrow discrete resonance and continuum. Fano resonance presents an obvious asymmetric shape, which can be expressed in the following formula [39]:

(1) $$I\propto \frac{{\left(F\gamma +\omega -\omega_{0}\right)}^{2}}{{\left(\omega -\omega_{0}\right)}^{2}+\gamma^{2}}$$

where *ω*_0_ represents the resonance frequency, *γ* is the width of the resonance, *F* stands for Fano parameter. According to Formula (1), the fitting results could be calculated which are shown in the inset of Figure 2. The values of *F* are both around 0.93 at two resonance frequencies.

**Figure 3:**
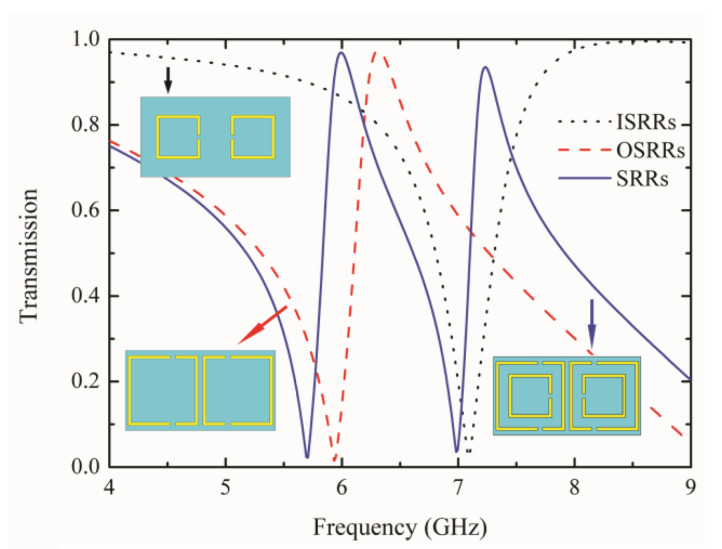
The transmission spectra of the OSRRs, ISRRs, and SRRs combined with the OSRRs and ISRRs.

In order to analyze the coupling mechanism among those SRRs, the transmission characteristics of different resonators (OSRRs, ISRRs, and SRRs) are displayed in Figure 3, respectively. The transmission curve of the OSRRs exhibits an obvious resonance at the frequency of 5.94 GHz, but becomes a flat curve at 7–8 GHz. When there are only ISRRs, the transmission spectrum is relatively smooth, and there is only one obvious valley at 7.1 GHz. Thus, dual-band Fano metamaterial could be realized by combining two types of SRRs. When the ISRRs are combined with the OSRRs, two Fano resonance peaks appear at 6 GHz and 7.23 GHz caused by the interference of the bright and dark modes. At 6 GHz, OSRRs and ISRRs generated the bright and dark modes, while the situation was reversed at the second Fano window.

**Figure 4:**
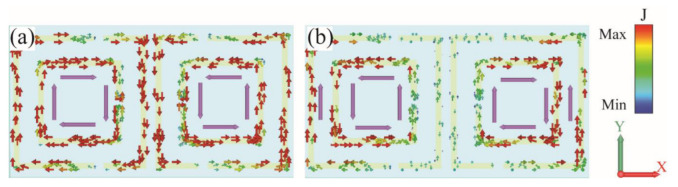
The distribution of the surface current at (**a**) 6 GHz and (**b**) 7.23 GHz.

The Fano resonances in toroidal metamaterial are excited by normal incident y-polarized electromagnetic waves. In order to clarify the resonance modes of the two Fano resonance peaks in Figure 2 and elaborate on the coupling process between the proposed SRRs, the surface current in the structure is studied by simulation, which is shown in Figure 4. At peak I, the reversed surface current loops appear on the SRRs, as indicated by the purple arrows in Figure 4a. The current flows, both inner and outer, along the clockwise and anti-clockwise direction in the left and right resonators of the unit cell. Obviously, such paired reversed current loops can form a pair of magnetic dipoles in opposite directions, which can lead to the enhanced toroidal dipole moment along the y-axis while suppressing the electric dipoles.

The surface current distribution of the second resonant mode at peak II is shown in Figure 4b. Unlike the first mode, the induced current on the OSRRs in this mode is very small, which is almost concentrated on the outermost non-split arms. Meanwhile, the ISRRs are strongly excited with a pair of current loops along opposite directions. The induced current of the ISRRs in the y-direction is partially offset by the OSRRs.

To demonstrate quantitatively that the dual-band Fano resonances are produced by toroidal dipole response, the scattered power of the multipoles is calculated by the conduction current density according to the multipole scattering theory. The multipole moment could be acquired by the following formula in the Cartesian coordinate system (α, β = x, y, z).

(2) $$P=\frac{1}{i\omega }\int jd^{3}r$$

(3) $$M=\frac{1}{2c}\int (r\times j)d^{3}r$$

(4) $$T=\frac{1}{10c}\int [(r\cdot j)r-2r^{2}j]d^{3}r$$

(5) $$Q_{e}=\frac{1}{i2\omega }\int [r_{\alpha }j_{\beta }+r_{\beta }j_{\alpha }-\frac{2}{3}(r\cdot j)\delta_{\alpha ,\beta }]d^{3}r$$

(6) $$Q_{m}=\frac{1}{3c}\int [{\left(r\times j\right)}_{\alpha }r_{\beta }+{\left(r\times j\right)}_{\beta }r_{\alpha }]d^{3}r$$

In the Formulas (2)–(6), **P** and **M** represent the conventional electric and magnetic dipole moments. **T** is the intriguing toroidal dipole moment. *Q*_e_ and *Q*_m_ are the electric and magnetic quadrupole moments. *c* is the speed of light, **j** is the induced current density, and **r** stands for the distance vector. The total multipoles scattered power can be calculated by Formula (7).

(7) $$I=\frac{2\omega^{4}}{3c^{3}}{\left|P\right|}^{2}+\frac{2\omega^{4}}{3c^{3}}{\left|M\right|}^{2}+\frac{2\omega^{6}}{3c^{5}}{\left|T\right|}^{2}+\frac{\omega^{6}}{5c^{5}}{\sum \left|Q_{e}\right|}^{2}+\frac{\omega^{6}}{20c^{5}}{\sum \left|Q_{m}\right|}^{2}+ο(\frac{1}{c^{5}})$$

**Figure 5:**
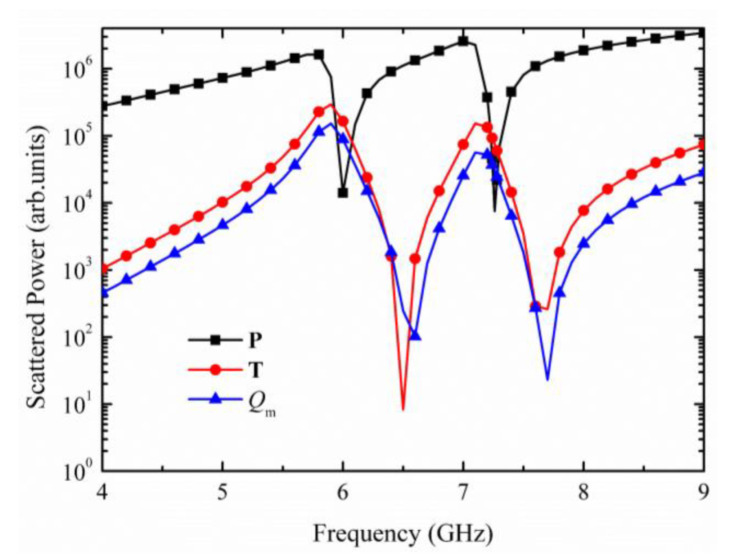
Scattered power of the three strongest multipole excitations (**P**, **T** and *Q*_m_).

The first five terms stand for the scattered power of **P**, **M**, **T**, *Q*_e_, and *Q*_m_, respectively. The last term is a higher-order correction, which can be ignored in our proposed subwavelength metamaterial. Figure 5 plots the scattered power of multipoles, and to obtain a clearer image, only the three strongest multipole excitations **P**, **T**, and *Q*_m_ have been exhibited.

The electric dipole has strong radiation loss. As shown in Figure 5, the scattered power of electric dipole has dominated almost the entire far-field scattered spectrum and emerged the maximum values at 5.7 GHz and 6.98 GHz, corresponding to the transmission dips. However, the electric dipole is restrained largely due to the multipole interactions in the metamaterial at the two resonant points and shows a sharp decreasing trend at 6 GHz and 7.23 GHz. Meanwhile, the scattered power of toroidal dipole increases significantly, and is approximately 10-times higher than the electric dipole. The dominating toroidal dipole far-field scattered power at resonance frequency further demonstrates that the dual-band Fano resonances are both toroidal dipole response. As a fancy property of toroidal, a large proportion of energy could be well concentrated inside the metamaterial instead of dissipating. Due to the current properties of the toroidal dipole, the magnetic quadrupole has the same tendency as toroidal dipole in this system, which inhibits the structure from producing a higher transmission coefficient at the two resonance points.

**Figure 6:**
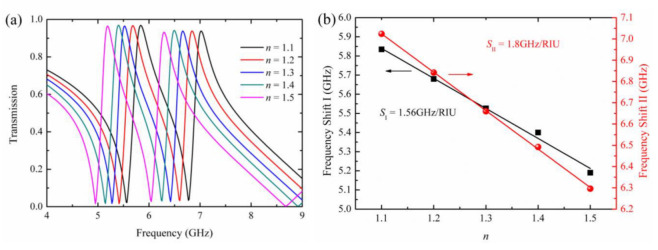
(**a**) The resonance frequency shift with the increase in the refractive index of analyte; (**b**) The linear fit of the frequency shift I (black line) and II (red line).

By introducing metamaterial, the change in the effective permittivity can be expressed in terms of the spectral shift. When the analyte is coated on the surface of toroidal metamaterial, the dielectric environment around the metamaterial changes, resulting in a variation of the effective permittivity. The sensitivity of the toroidal metamaterial can be calculated by Formula (8):

(8) $$S=\frac{\Delta f}{\Delta n}$$

where Δ*f* is the frequency shift of the transmission amplitude curve of the metamaterial, and Δ*n* is the change in the refractive index. With *n* increasing from 1.1 to 1.5, both transmission peaks of the toroidal metamaterial demonstrate an obvious red shift, as shown in Figure 6a. The linear fit is performed and displayed in Figure 6b. The sensitivity of the first and second transmission window, *S*_I_ and *S*_II_, are 1.56 GHz/RIU and 1.8 GHz/RIU, respectively. According to the characteristics of the toroidal dipole, the energy is concentrated in a small area around the structure, so the radiation loss of the structure is relatively low, and the analyte has a strong coupling with the incident waves.

**Figure 7:**
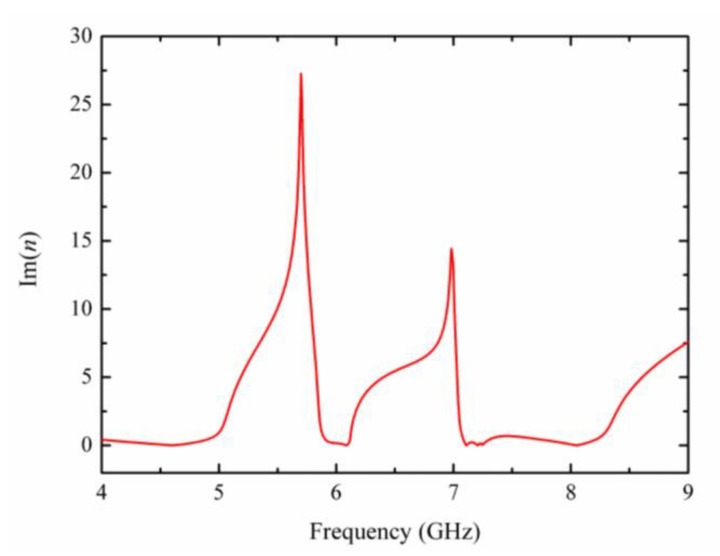
The imaginary part of effective refractive index of the proposed toroidal metamaterial.

The loss of the subwavelength structure is determined by the imaginary part of the effective refractive index, which can be calculated from the S-parameter according to the electromagnetic parameter retrieval method. As shown in Figure 7, the imaginary part of the effective refractive index of the toroidal metamaterial (Im(*n*)) has large values at 5.7 GHz and 6.98 GHz, so that the loss of the proposed metamaterial tends to peak, and the incident electromagnetic waves are strongly scattered. At peaks I and II, Im(*n*) are both almost close to zero, which indicates that the proposed toroidal dipole metamaterial has low-loss characteristics and results in high Fano peaks.

**Figure 8:**
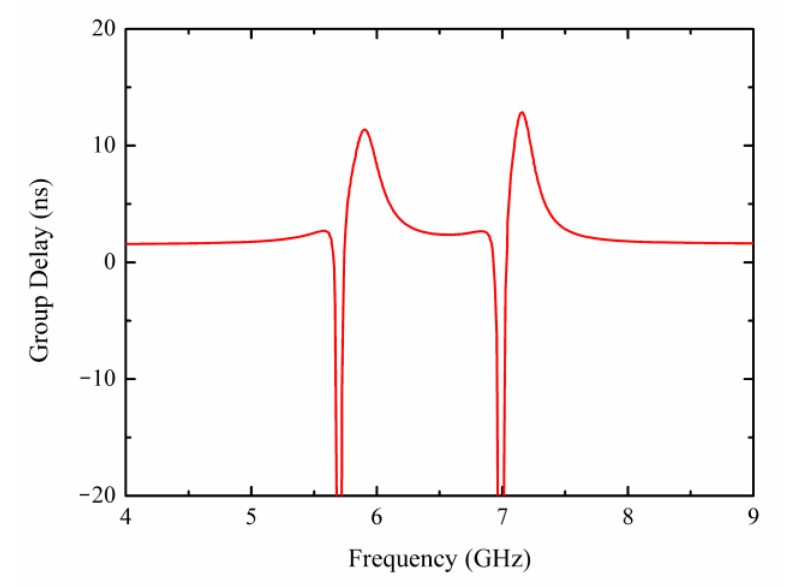
The group delay of the subwavelength structure.

The high-transmission sharp profile of the dual-band Fano resonances has the potential to achieve strong dispersion and slow light effects with a large group delay. This property of toroidal metamaterial can slow down the propagation speed of electromagnetic waves in media and effectively enhance the interaction between light and matter. The corresponding group delay (*τ*_g_) of the metamaterial in the Fano windows can be evaluated by the formula:

(9) $$\tau_{g}=-\frac{d\varphi (\omega )}{d\omega }$$

where *φ*(*ω*) denotes the phase shift of the transmission spectrum, and *ω* is the angular frequency of the resonance. It can be observed from Figure 8 that *τ*_g_ increases to 11.38 ns and 12.85 ns at 6 GHz and 7.23 GHz, which means that the speed of electromagnetic waves passing through the toroidal dipole metamaterial slows down at the two Fano resonance points. This low-loss dual-band Fano toroidal dipole metamaterial is potentially valuable in slow light and enhanced light–matter interaction applications.

## 4. Conclusions

In general, a dual-band Fano toroidal metamaterial with low-loss is proposed at microwave frequency. When the subwavelength metamaterial is irradiated by normal incident electromagnetic waves, the transmission coefficients reached 0.97 and 0.94 at two resonance peaks 6 GHz and 7.23 GHz, respectively. The dominant role of toroidal dipole at both Fano resonances could be demonstrated by the simulated current distribution and the calculated scattered power of far field. The presented toroidal metamaterial can realize the refractive index sensing with high sensitivity and also possesses slow light effects. This interesting dual-band toroidal metamaterial could have potential applications in ultra-sensitive sensors, filters, modulators, and slow light devices at microwave, terahertz, and optical frequencies.

## Data Availability

The data presented in this study are available on request from the corresponding author.
